# Professional Reputation

**Published:** 1856-11

**Authors:** 


*Professional Reputation.*

Dr. Baillie, of London, remarked that he had never known a physician, who, from any cause, acquired business rapidly in London, who permanently retained it. If it be rapidly acquired, this must be accomplished by means independent of those which give a firm hold on the confidence and affections of patients, for they cannot at once be displayed, nor can they at once have their full operation. Sir Astley Cooper's receipts from his first year's practice, were $26; the second year, $130; and so on, until on the ninth year it amounted to $5,500. Afterwards, his receipts ran up in one year to the enormous amount of $115,000. Dr. Hope, with a well-known London reputation, made $1,000 the first two years.—*Prof. Barker's* (*N.Y. Med. College*) *Introductory Lecture.*

---

☞ Dr. George C. Blackman, Professor of Surgery in the Medical College of Ohio, has become associated with Dr. T. Wood, also a Professor in the same College, in the proprietorship and editorship of the *Western Lancet.*—*Boston Med. and Surg. Journal.*

